# Total Antioxidant Capacity and Total Oxidative Capacity in Multi-Modal Opioid-Based Therapy for Non-Cancer Pain: Analysis of Redox Status

**DOI:** 10.3390/cimb48050437

**Published:** 2026-04-23

**Authors:** Urszula Kosciuczuk, Piotr Jakubow, Marcin Talalaj, Katarzyna Grabowska

**Affiliations:** Department of Anaesthesiology and Intensive Therapy of Children, Medical University of Bialystok, Kilinskiego Street 1, 15-089 Bialystok, Poland

**Keywords:** analgesics, chronic pain, oxidative stress

## Abstract

Current scientific reports on pain pharmacotherapy focus on the side effects of opioid medications related to dysregulation of the oxidative–antioxidant balance and immunomodulation. Initial observations concerned the use of opioids in the treatment of acute postoperative and cancer pain. Little is known about oxidative stress modulation in multi-modal opioid-based analgesia for chronic non-cancer pain. The aim of this study was to describe oxidative stress using plasma total antioxidant capacity (TAC) and total oxidative capacity (TOC), to assess whether these metrics are dependent on pain intensity and the scheme of analgesia. The study group consisted of patients with chronic low back pain, who were divided under the following treatments: multi-modal opioid-based therapy (n = 42), monotherapy with opioids (n = 28), and the control group (n = 11). A significantly lower TAC was observed in the study group compared to the monotherapy and control groups (220 µmol/L vs. 295 µmol/L, *p* = 0.02 vs. 399 µmol/L, *p* = 0.01). TOC was significantly lower in the polytherapy group compared to the monotherapy group (594 µmol/L vs. 723 µmol/L, *p* = 0.0002). A significantly lower TAC was observed in the typical analgesia scheme compared to the adjuvant analgesia model (260 µmol/L vs. 339 µmol/L, *p* = 0.01). The TAC in the severe pain classification was significantly lower than in the moderate group (*p* = 0.03). Multi-modal therapy with opioids significantly reduced oxidative activity compared to monotherapy but did not improve antioxidant capacity. Opioid-based pain therapy combined with adjuvant analgesics produced better antioxidant properties, and the antioxidant capacity was lower in severe pain scores.

## 1. Introduction

Current guidelines allow the use of opioid analgesics in cancer and non-cancer acute and chronic pain therapy as the second level of the analgesic ladder in moderate pain (weak opioids) and the third level of the analgesic ladder in severe pain. Opioid analgesics are mixed with typical analgesics (paracetamol, metamizole, nonsteroidal anti-inflammatory drugs, i.e., NSAIDs) and adjuvant analgesics to improve the analgesic effect and limit adverse reactions. LBP (low back pain) is the most frequent non-cancer chronic pain and has been reported as the most common indication for the use of opioid drugs both in in-hospital and out-of-hospital practice. Observational studies have described the dynamic growth in opioid therapy for LBP being from 19% to 29%. Furthermore, the crucial medical aspects of adverse reactions are associated with long-term use, and prolonged opioid therapy is significantly more common compared to nonsteroidal anti-inflammatory drugs (8.5% of patients continue opioid therapy within a year compared to 2% with NSAIDs) [1,2,3,4,5,6,7,8,9].

Due strong addictive properties and psychiatric effects, the use of opioid analgesics requires careful clinical supervision and assessment of the efficacy and benefits of the therapy. The great effectiveness of opioid drugs in pain therapy is based on the multi-directional effects upon the central and peripheral structures of the nociceptive pathways (primary C-fiber afferent neurons, neurons in the dorsal horn of the spinal cord, and central brain structures, ventral tegmental area, nucleus accumbens, periaqueductal gray, raphe magnus, and locus coeruleus) and the modulation of all stages of nociception (transduction, transmission, modulation, and perception) [10,11,12].

The mechanism of action of opioid drugs is both well understood and described. Cellular activity involves the activation of transmembrane G protein subunits; the activation of phospholipase C (PLC), adenyl cyclase (AC), and protein kinase A (PKA); the generation of intracellular transmitters leading to the production of 1,4,5 trisphosphate (IP3), which consequently causes changes in the permeability of cell membranes to chloride and potassium ion channels, and voltage-gated calcium channels. These mechanisms inhibit postsynaptic and presynaptic neuronal activity and nociception. Moreover, opioids modulate GABA (gamma-aminobutyric acid), NMDA (N-methyl-D-aspartate), and noradrenergic, as well as dopamine transmission [13,14,15,16,17,18,19,20].

Antioxidant–oxidative dysregulation is the newest aspects regarding the consequences of opioid-based therapy. Current knowledge indicates that this process is very complex and multi-directional, and most data are drawn from experimental studies. Opioid-based intracellular oxidative stress occurs via mitochondria (CREB cycles generate free electrons and radical sources, dysregulating NADPH oxidases), cytoplasmic (decreased activity of superoxide dismutase (SOD) and catalase (CAT), and nuclear mechanisms (NF-kB and HIF 1 alpha regulate the expression of pro-inflammatory and redox-signaling factors and enzymes). Moreover, the greatest oxidative stress imbalance is expressed by the activation of mi opioid receptors (MORs) [21,22,23].

In a previous study, we demonstrated that total antioxidant activity was significantly lower in the group of patients undergoing opioid monotherapy. At the same time, oxidative capacity was significantly lower for buprenorphine compared to oxycodone and tramadol. The highest antioxidant potential values were observed for oxycodone, while significantly lower values were reported for buprenorphine and tramadol. Considering the TOC/TAC ratio, the most favorable oxidative–antioxidant balance was observed for buprenorphine. Furthermore, our study did not demonstrate a relationship between the redox balance parameters of TAC and TOC and the duration of opioid therapy or dose [24,25,26].

The most pronounced clinical and practical effects of opioids on oxidant–antioxidant balance and immunomodulation have been described in relation to pain therapy in patients with cancer pain. Revolutionary studies have demonstrated that perioperative opioids influence the course of cancer and the progression of metastases. Therefore, restricting opioid use during anesthesia and the immediate postoperative period, along with the simultaneous use of multi-modal analgesia is recommended. Based on these findings, doubts arise regarding the use of opioids in the treatment of chronic non-cancer pain.

The results of experimental studies are insufficient for clinical conclusions. Furthermore, there are few publications regarding the effects of opioid substances on the markers of oxidative stress. Human opioid-mediated oxidative stress is poorly investigated. Previously, it has been reported that opioid administration changes the oxidative balance in the perioperative period of oncology surgeries and changes the methods of anesthesia during surgery, with limitations upon opioid administration. Secondly, little is known about oxidative stress in opioid-addicted patients. Moreover, there is scant knowledge regarding oxidative stress in chronic pharmacotherapy with opioids [27,28,29,30].

In this study, we hypothesized that multi-modal opioid-based non-cancer pain therapy models cause oxidative–antioxidant imbalances under different schemes compared to the opioid-monotherapy model. This study aimed to quantitatively describe the oxidative stress disturbances based on plasma total antioxidant activity (TAC) and plasma total oxidative activity (TOC). Additionally, we aimed to assess whether oxidative stress parameters depend on pain intensity.

## 2. Materials and Methods

This study was approved by the Ethics Committee of the Medical University of Bialystok, Poland (R-I-002/307/2019), registered at Clinical Trials.gov (NCT 04227223), and performed in accordance with the standards of the Declaration of Helsinki. Adult patients who met the criteria due to chronic low back pain and received opioid therapy as a polytherapy scheme were eligible for study inclusion. The WHO definition of chronic low back pain (LBP) describes pain between the lower edge of the ribs and the buttocks for a period exceeding 6 months. Exclusion criteria were as follows: advanced renal failure, metabolic disorders, diabetes, diabetes neuropathy, dysregulations of thyroid hormones, hypercholesterolaemia, cancer and postoperative pain, and antioxidant supplementation. In the first stage of this study, we analyzed the study group, comprising patients undergoing opioid monotherapy with a stable pain score, and the control group, comprising patients with LBP without any pharmacotherapy.

The procedure was fully explained to each participant, and all patients signed an informed consent. Then, the participants were surveyed for demographic information such as age, gender, and anthropometric parameters such as weight and height. Participants identified their most affected pain severity in the previous week, and actual pain severity was assessed using the NRS score in the range of 0–10. We have used the nomenclature of pain intensity based on International Pain Society recommendations. An NRS score of between 1 and 3 was reported as a mild pain, an NRS score of between 4 and 6 as moderate pain, and NRS values between 7 and 10 were assigned as severe pain. Medical information regarding pharmacotherapy was also recorded.

Recruitment was carried out in the morning. Patients took medications according to the prescribed schedule, and blood was collected while maintaining a food and fluid regime for at least 8 h.

Following clinical assessment, the researchers collected blood samples via venipuncture. Blood samples were aseptically drawn into ethylenediaminetetraacetic acid tubes (2.7 mL EDTA BD vacutainers). Within 30 min of collection, the samples were centrifuged (1000× *g* for 15 min at 2–8 °C) and immediately stored at −80 °C in aliquots of 300 microliters in Eppendorf tubes until required. Plasma TAC and TOC were analyzed using photometric methods (PerOX, ImAnOx, Immunodiagnostic AG, Bensheim, Germany) and calculated in µmol/L. Sample processing and data analysis were performed according to the manufacturer’s instructions (Immunodiagnostik, Bensheim, Germany).

The determination of the total antioxidative capacity (TAC, ImAnOx) was performer using the reaction of antioxidants in the sample with a defined amount of exogenously provided hydrogen peroxide (H_2_O_2_). The antioxidants in the sample eliminated a certain amount of the hydrogen peroxide provided. The residual H_2_O_2_ was determined photometrically using an enzymatic reaction that involved the conversion to a colored product. After the addition of a stop solution, the samples were measured at 450 nm in a microtiter plate reader. The quantification was performed using a delivered calibrator. The limit of the TAC detection was 130 μmol/L. The difference between the applied and measured peroxide concentration in a defined time period is proportional to the reactivity of the antioxidants of the sample (antioxidative capacity). The difference in the sample values with and without the enzyme is inversely proportional to the antioxidative capacity. To obtain the ΔOD, one subtracts the OD values of the samples without the enzymes from the OD values of the samples with the enzyme. The antioxidative capacity was calculated according to the following formula: antioxidative capacity [μmol/L] = 392 − (392 − calibrator concentration) × [ΔODsample/ΔOD calibrator]

Based on Immundiagnostik studies of EDTA plasma and serum of healthy persons, the following reference values were estimated: low antioxidative capacity, <280 μmol/L; middle antioxidative capacity, 280–320 μmol/L; high antioxidative capacity, >320 μmol/L; with a mean value of 305 μmol/L.

The PerOx test (TOC) measures the activity of lipid peroxides. The determination of the peroxides was performed using the reaction of a peroxidase with peroxides in the sample followed by the conversion to a colored product. Measurement 1 presents the initial absorption of the samples in the ELISA reader at 450 nm. Measurement 2 was performed immediately after the addition of the stop solution at 450 nm in the ELISA reader. The difference between measurements 1 and 2 was directly and linearly proportional to the peroxide content of the sample.

The reference ranges were as follows: EDTA-plasma < 200 μmol/L, low oxidative stress; 200–350 μmol/L, moderate oxidative stress; >350 μmol/L, high oxidative stress with linearity up to 800 μmol/L and a detection limit of 7 μmol/L [9].

Statistica 14.1 (Statsoft, Cracow, Poland) was used for all statistical analyses. A Shapiro–Wilk test was used to determine the normal distribution of continuous values; therefore, nonparametric methods were implemented. Quantitative data were presented as the median, minimum, maximum, and interquartile ranges, CI 95% ranges and raw data. A Mann–Whitney U test was applied to analyze quantitative values of TAC and TOC between groups. The Kruskal–Wallis test was used to compare anthropometric parameters, TAC and TOC values between analgesic groups and pain intensity groups. A Spearman’s test was used to compare TAC and TOC values and the pain severity score in the different models of therapy. Statistical significance was established at *p* < 0.05. Data from our previous study were used to compare the characteristic parameters, the TAC and TOC values between groups.

The statistical analysis used data from a pilot study in which the initial study group size was set at 35 patients. Retrospective power analysis for the group size (n = 28 for study monotherapy group/n = 11 for control group) and the TAC and TOC values indicated a test power of 1.0 with an alpha error of 0.05. To assess the group size, a mean values difference of 15 for TAC and TOC was used, indicating a required group size of n = 11 while maintaining an alpha of 0.05 and a test power of 0.9. Recruitment was arbitrary, and the number of subsequent groups was limited by the availability of medical data.

## 3. Results

Of the 81 patients enrolled in this study, 42 were included in the polytherapy group. The data from the polytherapy group did not differ in the characteristic parameters (Table 1).

This group consisted of 18 patients under a typical analgesia scheme and 24 patients under an adjuvant analgesia scheme. The most popular of the typical analgesia schemes consisted of a combination of opioid/paracetamol/diclofenac and was noted in 35% patients. Additionally, a combination of opioid/pregabalin/steroids was noted in 45% of patients under an adjuvant analgesia scheme. A mild pain severity score (NRS 1–3) was reported in 19 patients, a moderate pain severity score (NRS 4–6) in 14 patients, and a severe pain score (NRS 7–10) in 9 patients in the multimodal opioid model. Ten patients from the study group reported sleep disorders, six patients underwent active rehabilitation at the time of recruitment, and fifteen patients declared that they exercised regularly. The chronic nicotinism was reported in 36 patients.

In the polytherapy group with opioids, significantly lower TAC values compared to the group of patients with monotherapy (220 µmol/L vs. 295 µmol/L, *p* = 0.02) and the control group (220 µmol/L vs. 399 µmol/L, *p* = 0.01) were observed. TOC values were significantly lower in the polytherapy group compared to the opioid monotherapy (594 µmol/L vs. 723 µmol/L, *p* = 0.0002); however, no significant difference was observed compared to the control group (594 µmol/L vs. 533 µmol/L) (Table 2).

**Figure 1:**
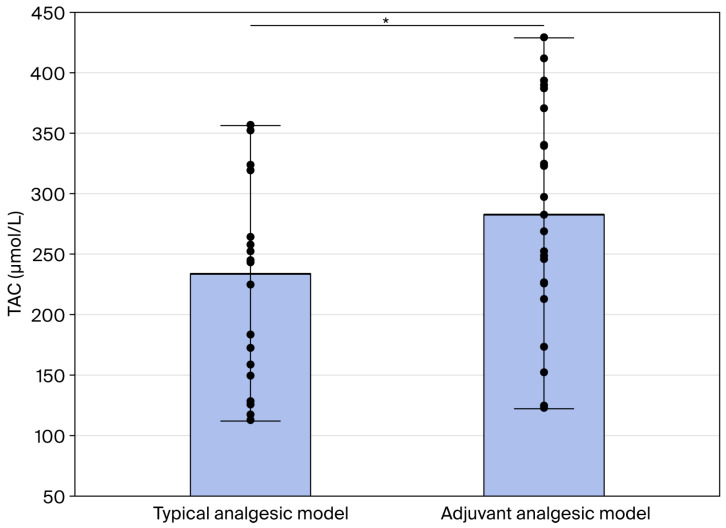
The TAC values in typical and adjuvant analgesic model. The median, minimum–maximum ranges and raw data are presented. * Statistical significance with *p* < 0.05.

The TAC and TOC values did not differ between patients with and without nicotinism in multimodal opioid-based therapy. In the group of patients with multi-modal opioid-based analgesia under a typical analgesic model, significantly lower median TAC values were observed, with a median value of 260 µmol/L compared to the 339 µmol/L, *p* = 0.01, of the adjuvant analgesic model (Figure 1).

There were no significant differences in TOC values between the typical and adjuvant analgesic models (Table 3).

**Figure 2:**
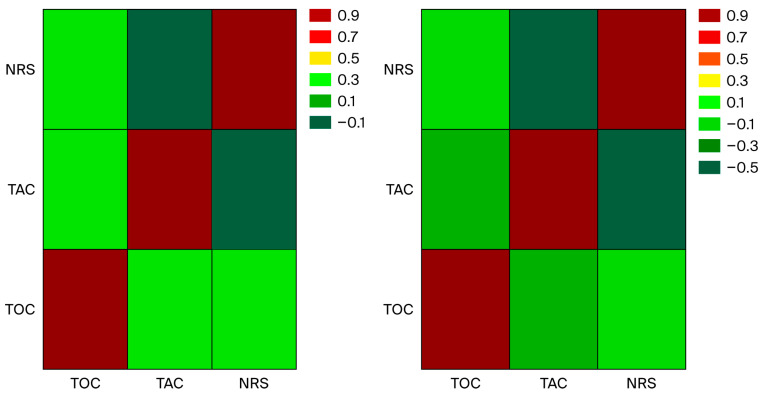
Heatmap of Spearman correlations in typical (**left**) and adjuvant (**right**) analgesic model.

Spearman’s test presented a significant negative correlation between TAC values and NRS score in the adjuvant analgesic model, with Rho −0.51, *p* < 0.05. The heat maps of the correlations in the typical and adjuvant analgesic models are presented in Figure 2.

The highest values of TAC were reported in the moderate pain score, but these results were not statistically significant compared to the mild pain score. The highest oxidant properties based on TOC values were noted in the severe pain score (Table 4).

**Figure 3:**
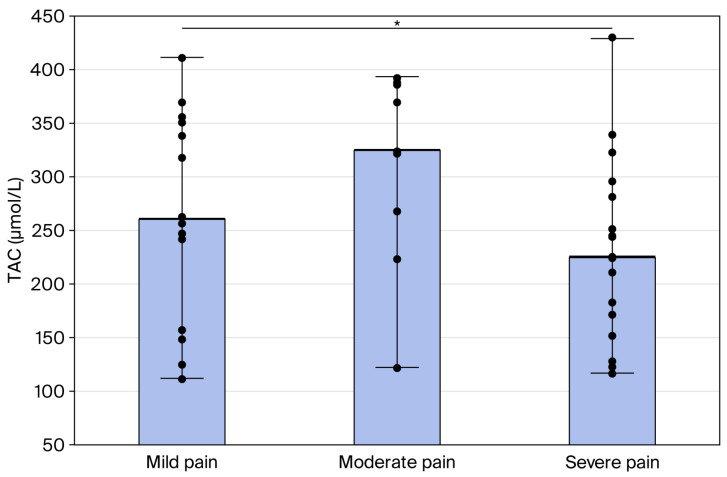
The TAC values in mild (1), moderate (2), and strong pain (3). The median, minimum–maximum ranges and raw data are presented. * Statistical significance with *p* < 0.05 compared with moderate score.

The TAC values in the severe pain score were significantly lower than in the moderate-pain-score group (*p* = 0.03) (Figure 3).

## 4. Discussion

In our study, we analyzed the oxidative–antioxidant balance in patients using a multi-modal opioid-based pain therapy for chronic LBP. We demonstrated that this model of analgesic therapy reduces antioxidant capacity and oxidative activity compared to opioid monotherapy. Additionally, our study revealed that the typical analgesic model reduced antioxidant capacity more than the adjuvant analgesic model. In severe pain, antioxidant properties had the lowest median values, which were significantly lower compared to moderate pain.

Based on the available literature, little is known about the clinical correlations between chronic and acute pain treatment and oxidative stress biomarkers. Serum TAC was statistically lower in patients with chronic pain in general joint hypermobility, assessed at 1.62 µmol/L vs. 4.85 µmol/L, while serum TOC was significantly higher, 48.5 µmol/L vs. 23.8 µmol/L [31]. Similarly, oxidative stress as a biomarker is also common in fibromyalgia. The mean plasma total antioxidant capacity was 1.5 mmol/L, statistically lower than the control mean value, which was 1 mmol/L, while total peroxidase was higher than controls (37.4 µmol H_2_O_2_/L vs. 33 µmol H_2_O_2_/L). Moreover, an important inverse correlation between TAC and pain intensity based on VAS was noted, with rho −0.79 and *p* < 0.001 [32]. Plasma TAC was significantly lower in patients with rheumatoid arthritis compared to healthy individuals, as well as osteoarthritis, with values of 1.10 vs. 1.43 vs. 1.44 mmol/L, while oxidative stress based on MDA was significantly higher in rheumatoid patients compared to controls and osteoarthritis (2.09 µmol/L vs. 1.13 vs. 1.24 µmol/L). Furthermore, significant negative correlations between MDA and TAC were found, with rho −0.398, *p* = 0.042. Additionally, a significant negative correlation was described between erythrocyte sedimentation rate and TAC, with rho −0.422 and *p* = 0.018 [33,34]. Oxidative stress biomarkers, such as plasma isoprostanes and isofluranes, preoperatively correlated with the worst pain intensity (beta 0.481, *p* = 0.04) and the McGill Pain Questionnaire Short Form scale MPQ (*p* = 0.001, beta 0.127) after 6 months following total knee arthroplasty. The plasma isoprostanes and isofluranes obtained intraoperatively were also presented as risk factors of the worst NRS and average NRS and MPQ values, *p* = 0.04, beta 0.230 vs. *p* = 0.02, beta 0.319 vs. *p* = 0.002, beta 0.114, respectively [35].

Another study suggested that oxidative stress based on plasma TOS was an independent predictor of surgical abdominal pain with an odds ratio of 1.163, *p* = 0.001. Moreover, plasma TOS level was noted as an important predictor of non-specific abdominal pain, with an odds ratio of 1.541, *p* = 0.001. TAC plasma was not significantly different in abdominal pain patients (1.09 mmol/L vs. 1.05 mmol/L), while TOS plasma was significantly higher in the abdominal pain group at 34.37 µmol H_2_O_2_/L vs. 25.73 µmol H_2_O_2_/L [36,37].

The parameters of oxidative stress based on superoxide dismutase (SOD) and glutathione peroxidase (GPx) also correlated with inflammatory markers (IL-1 and IL-6, C-reactive protein) in the preoperative and postoperative period. The factors that correlated with SOD activity on postoperative day 1 were high pain intensity and receiving opioids—tramadol on postoperative day 1. Receiving tramadol on the first postoperative day also correlated with high values of SOD on postoperative day 7. The oxidative biomarker did not correlate with pain intensity and pain pharmacotherapy [38]. As a part of opioid-based general anesthesia in oncology surgery, opioids presented significantly higher postoperative values of IL-12 compared to opioid-free anesthesia. Plasma antioxidant capacity was augmented after anesthesia, regardless of the use of opioids [39]. It was also observed that buprenorphine could suppress inducible NO synthetase activated by sepsis, but buprenorphine did not significantly change NO production compared to naloxone [40].

Relatively, most publications consider oxidative stress in opioid-addicted patients. Heroin-addicted patients present an impairment of the oxidative status of erythrocytes, with significantly higher levels of serum methemoglobin (MetHb), serum glutathione peroxidase (GPx), GPx activity, disulfide (SS), and native thiol (SH) among addicts. Furthermore, a significant association between MetHb and GPx activity has been observed, together with a higher concentration of erythrocytic protein carbonyl contents and GSSG/GSH (oxidized to reduced glutathione ratio) [41,42]. Opioid-dependent patients have presented an altered purine metabolism and increased quinine and xanthosine concentration with decreased quanosine and hypoxanthine, and hypoxanthine/xanthine and xanthine/xanthosine ratios. After detoxification with methadone, higher plasma levels of alpha and gamma tocopherol were recorded, and the GSH/GSSG ratio increased [43]. Impaired intracellular and extracellular homeostasis based on reduced GSH/GSSG and SS/SH were also noticed in opioid-dependent patients. Moreover, a positive correlation was found between SS, SS/SH%, SS/SH + SS%, GSSG, GSSG/GSH%, GSSG/GSH + GSSG%, and the duration and amount of opioid doses [43]. Opioid-dependent patients presented statistically higher values of catalase activity, 162 U/mL, compared to healthy controls (46 U/mL) and opioid withdrawal (87 U/mL). Opioid withdrawal patients also presented lower values of GPX (104 U) compared to controls (465 U, *p* < 0.01) and opioid-dependent patients (435 U, *p* < 0.01). SOD activity did not differ between opioid-dependent/opioid withdrawal patients and controls (30 U/mL vs. 26 U/mL vs. 27 U/mL), but it was significant between opioid withdrawal and opioid-dependent patients (*p* = 0.023) [44]. Exposure to opioid substances has been shown to reduce antioxidant capacity expressed as SOD and CAT activity in human erythrocytes, plasma, and organ tissues, primarily in hepatocytes and brain structures, such as the cerebrum and hippocampus. It was also noted that in people addicted to opioid substances, reduced plasma concentrations of antioxidant cofactors such as copper, selenium, and zinc were observed [45,46].

The mechanisms of opioid-based oxidative–antioxidant dysregulation are highly complex. Firstly, the cellular physiology of MOR activation induces mitochondrial, cytoplasmatic, and nuclear oxidative activity. Secondly, the crucial mechanisms of opioid-induced oxidative stress are connected with metabolism and the cytochrome P450 system, in particular CYP3A4 and CYP2D6, for substances such as codeine, hydrocodone, oxycodone, fentanyl, methadone, and tramadol. The o-demethylation of oxycodone to oxymorphone, hydrocodone to hydromorphone, and codeine to morphine via CYP2D6 exposes tyramine-like phenol structures, which are subsequently transformed into oxidative radicals. Derivative metabolites of morphine are conjugated with glutathione in hepatocytes, which reduces the supply of this antioxidant. Moreover, opioid-induced cellular and humoral immunomodulation increases oxidative imbalance [47,48,49,50].

In our study, we assessed the redox balance in complex opioid-based polytherapy with typical and adjuvant analgesics. We did not find any differences in plasma TOC between typical and adjuvant analgesia models, but plasma TAC values were higher in the adjuvant analgesic model. This means that this scheme of chronic pain therapy improved antioxidant properties. The typical analgesics present direct activity in oxidative disturbances. The hepatocellular metabolism of paracetamol reduces antioxidant capacity by utilizing glutathione resources and increases oxidative potential, which is expressed by increased MDA concentrations. NSAIDs modulate the inflammatory response and inhibit the natural function of platelets, displaying a greater anti-inflammatory potency than acetaminophen. Aspirin decreases the levels of reactive species in human hepatic cells but also increases the amount of reactive oxygen species in adipocytes, gastric, and intestinal cells. Moreover, steroids, as popular adjuvant analgesics, exhibit strong anti-inflammatory and immunomodulatory effects [48,49,50,51,52].

Pain perception is a subjective process, and many endogenous and exogenous factors influence the mechanisms involved. Sex differences, lifestyle factors, metabolic factors, and dietary factors, antioxidant supplementation have been described [20,21,53,54,55,56,57,58,59,60]. In our study, we recruited patients of a similar age, with a similar number of males and females. Furthermore, we did not try to compare oxidative stress parameters with nutritional status or a functional frailty score. Oxidative stress assays using TAC and TOC are not standard laboratory or clinical procedures. Currently, they are the latest scientific trend. The biochemical parameters of organ function are indirect indicators of systemic oxidative stress in clinical practice [61,62,63,64,65].

The main limitation of our study is the small sample size. Study group was limited by number of patients including those receiving opioid medications. However, it should be emphasized that the use of opioid drugs as an element of chronic pain therapy requires an analysis of their benefits and risks. Moreover, it is not a first-line therapy, and the number of patients undergoing opioid monotherapy is very limited, and recruitment is difficult. Furthermore, the polytherapy regimens were so diverse that it was difficult to analyze individual models, making it impossible to assess the synergistic effects of various analgesics. Additionally, it is known that other biological, sociological, and psychological factors, as well as rehabilitation activities, also influence pain perception. We did not consider these factors in our analysis. Interpretation of the statistical results should be cautious due to the small sample size. Despite differences in the absolute values of individual parameters between groups, statistical analyses did not reveal significant differences (ex. TOC). Furthermore, the study group is characterized by high variability in clinical characteristics and a variety of analgesic regimens combining opioids with co-analgesics and adjuvant medications. Further study, analysis and clinical interpretation of the results require studies with a larger sample size.

Due to the limitations of this study, it is necessary to confirm the results in a larger cohort before we can adequately answer the research question. As an extension of this study, there is a need to examine the other effects of the long-term use of opioids, including their impact on cellular biomarkers of oxidative status.

## 5. Conclusions

The use of opioids in the treatment of chronic LBPs increases oxidative activity. However, multi-modal opioid-based analgesia in chronic LBP significantly reduced oxidative activity compared to opioid monotherapy. Additionally, in the opioid therapy of chronic LBPs, antioxidant activity was lower compared to the control group, and the multi-modal opioid analgesia caused a greater antioxidant deficit compared to opioid monotherapy. The adjuvant analgesic model presented better antioxidant properties without any significant oxidative changes. The highest values of oxidative stress and the lowest antioxidant activity were noted in severe pain treated with a multi-modal opioid scheme. The most common dysregulation of oxidative–antioxidant status was in cases of severe pain.

These results may alter the clinical models of therapy. Opioid-based analgesia mixed with adjuvant or typical analgesics reduces the risk of oxidative stress dysregulation. Adjuvant analgesics should be mixed with opioids because they increase the protective mechanism of antioxidation.

## Figures and Tables

**Table 1:** Characteristic of the patients included in the study.

Parameter	Multi-Modal Opioid-Based Analgesia	Opioid-Based Monoanalgesia	Control Group
Age [median, min.–max. ranges]	72.6 (47–76)	70.5 (43–80)	64.0 (30–81)
Sex n, female/male	42 (20/22)	28 (10/18)	11 (6/5)
BMI [median, min.–max. ranges]	24.8 (19.7–31.3)	27.2 (21.2–36.3)	26.7 (21.3–34.6)

**Table 2:** The TAC, TOC in polytherapy, monotherapy, and control group. The median and IQR, and CI 95% ranges are presented.

	Multi-Modal Opioid-Based Analgesia	Opioid-Based Monoanalgesia	Control Group
Total antioxidative capacity TAC (µmol/L)	220	295	399
	150–368 †,#	235–335 *	393–782
	119–320	180–306	286–640
Total oxidative capacity TOC (µmol/L)	594	723	533
	310–1124 †	391–1253	455–1480
	284–1062	352–1161	394–1240

* Statistical significance with *p* < 0.05, monotherapy compared to controls. † Statistical significance with *p* < 0.05, polytherapy compared to monotherapy. # Statistical significance with *p* < 0.05, polytherapy compared to controls.

**Table 3:** TAC and TOC in typical and adjuvant analgesic models. The median and IQR ranges, and CI 95% ranges are presented.

	Typical Analgesic Model	Adjuvant Analgesic Model
Total antioxidative capacity TAC (µmol/L)	236	285
	150–261	227–368
	138–252	160–354
Total oxidative capacity TOC (µmol/L)	384	468
	290–1050	315–1265
	262–992	285–1058

**Table 4:** TAC and TOC in pain severity categories based on NRS. The median and IQR ranges, and CI 95% ranges are presented.

	Mild Pain NRS 1–3	Moderate Pain NRS 4–6	Severe Pain NRS 7–10
Total antioxidative capacity TAC (µmol/L)	260.4	324.5	225.3
	157.9–351.6	268.4–386.7	162.3–267.3
	119–320	180–306	286–640
Total oxidative capacity TOC (µmol/L)	571.4	552.4	598.5
	381.2–1106.5	406.3–994.8	450.5–1260.4
	284–1062	352–1161	394–1240

## Data Availability

Data are unavailable due to privacy or ethical restrictions.
